# Determination of the Optimal Cut‐Off Point of Anthropometric Indices to Predict the Risk of Metabolic Syndrome in Iranian Adult Population With Type 2 Diabetes Mellitus: A Cross‐Sectional‐Analytical Study

**DOI:** 10.1002/edm2.70231

**Published:** 2026-05-15

**Authors:** Mohammad Moqaddasi Amiri, Hadi Bazyar, Mohammad Reza Amini, Vahideh Aghamohammadi, Ahmad Zare Javid

**Affiliations:** ^1^ Department of Public Health Sirjan School of Medical Sciences Sirjan Iran; ^2^ Student Research Committee Sirjan School of Medical Sciences Sirjan Iran; ^3^ Nutrition and Food Security Research Center Isfahan University of Medical Sciences Isfahan Iran; ^4^ Department of Nutrition Khalkhal University of Medical Sciences Khalkhal Iran; ^5^ Faculty of Health and Life Sciences Northumbria University Newcastle upon Tyne UK

**Keywords:** anthropometric indices, cut‐off point, metabolic syndrome, type 2 diabetes mellitus

## Abstract

**Aims:**

Metabolic syndrome (MetS) and type 2 diabetes mellitus (T2DM) are two diseases that are related to each other and the presence of both can increase the risk of heart attack and death. The present study aimed to assess the efficacy of anthropometric indices in predicting the risk of MetS among T2DM patients in Iran.

**Materials and Methods:**

In this study, 400 T2DM subjects were included via convenience sampling. Some anthropometric information, such as weight, height, waist circumference, and hip circumference along with biochemical data including fasting blood sugar, lipid profile components, systolic and diastolic blood pressure, were collected. The performance of the anthropometric indices in predicting MetS was evaluated using receiver operating characteristic (ROC) curve analysis and estimation of the area under the curve (AUC) values.

**Results:**

Abdominal volume index (AVI) had the largest AUC in the total population. Although this result was also obtained in females, the highest value of AUC was related to vaisceral adiposity index (VAI) in males. All of the anthropometric indices increased the odds of MetS significantly, with *p* < 0.001 in all crude and adjusted models. Although AVI had the highest odds ratio in the crude model (21.28, 95% CI 12.26–36.92), the highest odds ratio belonged to Relative fat mass (RFM) in the adjusted models.

**Conclusions:**

All anthropometric indices used in the study were effective in predicting the odds of MetS. AVI and VAI showed the strongest associations with MetS in both genders. Given the cross‐sectional design of the study, these results should be interpreted as associations rather than causal or predictive relationships.

## Graphical abstract

Anthropometric indices are effective markers for MetS risk in T2DM patients.

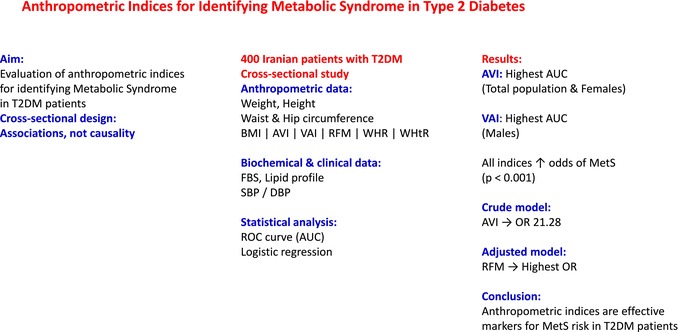

## 1 Introduction

Metabolic syndrome (MetS) is defined as a cluster of interrelated features characterised by obesity, hypertension, hypertriglyceridemia, low HDL‐C levels, and glucose intolerance, and increases the risk of type 2 diabetes mellitus (T2DM), ischemic heart disease, and stroke [1, 2]. The global prevalence of MetS has been reported to range from 38.3% to 47.6%, based on various criteria used to define it. In Iran, its prevalence varies from 32% to 47.6% based on different criteria. The estimated prevalence of this syndrome among Iranians in 2021 was 7.6 to 10.5 million for men and 7.1 to 12.7 million for women, respectively [3]. Currently, diabetes is one of the most significant health issues worldwide. According to the World Health Organization (WHO) in 2019, more than 460 million people worldwide have been diagnosed with diabetes, and this number is predicted to increase to more than 570 million by 2030 [4]. About 14% of the adult population in Iran has T2DM, and this rate is increasing [5, 6]. Between 65% and 73% of people with T2DM also show features of metabolic syndrome [7, 8]. The combination of high rates of diabetes and metabolic abnormalities increases the risk of cardiovascular disease and other related complications, which are currently the leading causes of death in Iran [9, 10].

MetS is closely associated with T2DM. The co‐occurrence of both diseases increases the risk of severe cardiovascular outcomes, kidney disease, and premature mortality [7, 11]. Early identification and risk prediction of metabolic syndrome in people with type 2 diabetes is crucial to prevent the onset of further complications. Traditional anthropometric indices, such as body mass index (BMI) and waist circumference (WC), have been widely used to assess obesity and predict the risk of metabolic syndrome [12, 13, 14, 15]. However, these indices have limitations, especially in populations with different ethnicities or conditions such as T2DM, where the distribution of fat and its association with metabolic risk factors may differ.

Novel anthropometric indices, such as waist‐to‐height ratio (WHtR), waist‐to‐hip ratio (WHR), a body shape index (ABSI), visceral adiposity index (VAI), abdominal volume index (AVI), and body adiposity index (BAI), have been found to predict the risk of MetS [16, 17, 18]. While the potential of these new indices suggests further use and applicability in routine clinical conditions, disagreement persists regarding the best cut‐off values, especially in populations such as those of Iranians, where genetic and environmental factors play a role in the relation between anthropometric measures and MetS risk. Recognition of those thresholds would be important to refine the care of patients with T2DM and for precision risk prediction.

Some studies have reported that the predictive value of these anthropometric indices could be quite different across populations due to genetic predisposition, body fat pattern, and lifestyle factors [19, 20]. In the Iranian setting, only a number of studies have examined the efficacy of conventional anthropometric indices in predicting MetS, and few studies have investigated the performance of new indices, specifically for individuals with T2DM [14, 21]. Secondly, the determination of population‐specific cut‐off points for the application of these indices is also an under‐explored area, restricting the clinical application of these measurements in real‐life practice. This study aims to determine the optimal cut‐off values of various anthropometric indices (WHtR, WHR, BAI, etc.) for MetS risk prediction in Iranian adults with T2DM. Through establishing Iranian‐specific cut‐off values for novel anthropometric indices in patients with T2DM, we hope to enhance the accuracy of risk stratification, facilitate earlier detection of high‐risk individuals, and ultimately promote more effective prevention and management strategies for MetS and T2DM in Iran.

## 2 Materials and Methods

### 2.1 Study Design and Participants

In this cross‐sectional study, 400 patients with T2DM who were referred to the Endocrinology and Metabolism Clinic of Golestan Hospital, located in Ahvaz, were included in the study between March and May 2023 using a convenient consecutive sampling method. Willingness to participate, age between 18 and 60 years, and at least 2 years have passed since T2DM diagnosis were the inclusion criteria. Exclusion criteria comprised insulin usage, pregnancy or lactation, smoking, alcohol consumption, incomplete demographic or anthropometric data, adherence to specialised diets, recent intake of antioxidant supplements within the last 3 months, and presence of comorbidities such as renal, hepatic, thyroidal, neoplastic, human immunodeficiency virus (HIV), or infectious diseases.

A questionnaire including demographic and anthropometric information (height in centimetres, weight in kilograms, waist circumference in centimetres, hip circumference in centimetres, gender, age in years, education, marital status, occupation, and medication history) and biochemical information (fasting blood sugar, lipid profile components, systolic and diastolic blood pressure) was used for data collection.

Waist circumference was measured from the upper part of the iliac crest and below the lower rib, and hip circumference was measured from the most prominent part of the hip using a tape measure. Fasting blood sugar and lipid profiles were extracted from the blood tests of patients under the supervision of a doctor in the medical diagnosis laboratory. Serum levels of fasting blood sugar and lipid profile were measured using Pars Azmoun kits using the enzymatic method. Glycosylated haemoglobin was obtained using the high‐performance liquid chromatography (HPLC) method and a Ds5 device. Also, whole blood samples were used to measure haemoglobin. All laboratory analyses were performed in a single certified laboratory using standardised assays, which minimised measurement variability. These procedures collectively enhance the reliability and internal validity of the study.

The protocol of this study was in accordance with the principles outlined in the Declaration of Helsinki and was approved by the Research Ethics Committee of Sirjan University of Medical Sciences (Ethical Code: IR.SIRUMS.REC.1403.028). Written informed consent was obtained from all participants before participating in the study.

### 2.2 Definition of MetS

According to the International Diabetes Federation (IDF) criteria, metabolic syndrome is defined as the presence of central obesity or a waist circumference of greater than or equal to 95 cm for both genders based on guidelines of the National Obesity Committee of Iran [22], in addition to two or more of the following four factors: triglycerides greater than or equal to 150 mg/dL, decreased High‐density lipoprotein (HDL) less than 40 mg/dL in men and less than 50 mg/dL in women, increased systolic blood pressure (SBP) greater than or equal to 130 with diastolic blood pressure (DBP) greater than or equal to 85 mmHg or drug treatment for blood pressure, impaired fasting glucose greater than or equal to 100 mg/dL or drug treatment for diabetes [1, 22].

### 2.3 Blood Pressure (BP) Measurement

Blood pressure (BP) was measured by a trained professional between 8:00 and 9:00 after 20 min of patient rest. Measurements were taken every 20 min, and the average of three consecutive values was recorded. Pulse pressure (PP) and mean arterial pressure (MAP) were calculated using the following formulas [23].

$$\mathrm{PP}\;\left(\text{mmHg}\right)=\mathrm{SBP}-\mathrm{DBP}$$

$$\mathrm{MAP}\;\left(\text{mmHg}\right)=\left[\mathrm{SBP}+\left(2\times \mathrm{DBP}\right)\right]/3$$

### 2.4 Anthropometric Indices Measurement

In this study, weight is measured without shoes and with as little clothing as possible while fasting using a body composition monitor digital scale made in Japan with an accuracy of 0.1. A tape measure with an accuracy of 0.5 cm will be used to measure height, with the subjects fully attached to the wall. BMI is calculated based on the formula of weight in kilograms divided by the square of height in meters [24].

WC (the distance between the iliac crest and the rib at the end of exhalation) and HC (at the level of the widest circumference over the greater trochanters) were measured using a non‐elastic tape.

The anthropometric indices were calculated using the following equations [12, 15, 21].

$$\text{Body mass index}\;\left(\mathrm{BMI}\right)=\text{weight}\;\left(\mathrm{kg}\right)/\text{height}\;{\left(m\right)}^{2}$$

$$\text{Triponderal mass index}\;\left(\mathrm{TMI}\right)=\text{weight}\;\left(\mathrm{kg}\right)/\text{height}\;{\left(m\right)}^{3}$$

$$\text{Waist to}\;\mathrm{hip}\;\text{ratio}\;\left(\mathrm{WHR}\right)=\mathrm{WC}\;\left(\mathrm{cm}\right)/\mathrm{HC}\;\left(\mathrm{cm}\right)$$

$$\text{Waist to height ratio}\;\left(\text{WHtR}\right)=\mathrm{WC}\;\left(\mathrm{cm}\right)/\text{height}\;\left(\mathrm{cm}\right)$$

$$\text{Body adiposity index}\;\left(\mathrm{BAI}\right)=\left[\mathrm{HC}\;\left(\mathrm{cm}\right)/\text{height}\;{\left(m\right)}^{1.5}\right]-18$$

$$A\;\text{body shape index}\;\left(\text{ABSI}\right)=\mathrm{WC}\;\left(m\right)/\left[{\mathrm{BMI}}^{2/3}\;\left(\mathrm{kg}/m^{2}\right)\;{\text{height}}^{1/2}\;\left(m\right)\right]$$

$$\begin{array}{cc} \text{Visceral adiposity index}\;\left(\mathrm{VAI}\right)\; & =\left(\mathrm{WC}\;\left(\mathrm{cm}\right)/\left[39.68+\left(1.88\times \mathrm{BMI}\right)\right]\right)\\ & \times \left(\mathrm{TG}\;\left(\text{mmol}/L\right)/1.03\right)\\ & \times \left(1.31/\mathrm{HDL}\;\left(\text{mmol}/L\right)\right)\;\text{in males} \end{array}$$

$$\begin{array}{cc} \text{Visceral adiposity index}\;\left(\mathrm{VAI}\right)\; & =\left(\mathrm{WC}\;\left(\mathrm{cm}\right)/\left[36.58+\left(1.89\times \mathrm{BMI}\right)\right]\right)\\ & \times \left(\mathrm{TG}\;\left(\text{mmol}/L\right)/0.81\right)\\ & \times \left(1.52/\mathrm{HDL}\;\left(\text{mmol}/L\right)\right)\;\text{in females} \end{array}$$

$$\text{Weight}-\text{adjusted waist index}\;\left(\mathrm{WWI}\right)=\mathrm{WC}\;\left(\mathrm{cm}\right)/\text{Weight}\;{\left(\mathrm{kg}\right)}^{2}$$

$$\text{Conicity index}\;\left(\mathrm{CI}\right)=\mathrm{WC}\;\left(m\right)/\left[0.109\times \sqrt{\text{weight}\frac{\mathrm{kg}}{\text{Height}}\left(m\right)}\right]$$

$$\begin{array}{cc} \text{Body roundness index}\;\left(\mathrm{BRI}\right)\; & =364.2-365.5\\ & \times {\left(1–{\left[\mathrm{WC}/2\pi \right]}^{2}/{\left[0.5\times \text{height}\right]}^{2}\right)}^{½} \end{array}$$

$$\begin{array}{c} \text{Relative}\;\mathrm{fat}\;\text{mass}\;\left(\mathrm{RFM}\right)=64-\left(20\times \text{height}/\mathrm{WC}\;\left(\mathrm{cm}\right)\right)\\ +\left(12\times \mathrm{sex}\right);\mathrm{sex}=0\;\text{for males and}\;1\;\text{for females} \end{array}$$

$$\begin{array}{c} \text{Abdominal volume index}\;\left(\mathrm{AVI}\right)\\ =\left[2\times \mathrm{WC}\;{\left(\mathrm{cm}\right)}^{2}+0.7\times {\left(\mathrm{WC}\;\left(\mathrm{cm}\right)-\mathrm{HC}\;\left(\mathrm{cm}\right)\right)}^{2}\right]/1000 \end{array}$$

$$\begin{array}{cc} \left(\mathrm{mg}/\mathrm{dL}\right)\;\text{for}\;\mathrm{TG}\; & =\left(\text{mmol}/L\right)\times 88.5\;and\;\left(\mathrm{mg}/\mathrm{dL}\right)\;\text{for}\;\mathrm{HDL}\\ & =\left(\text{mmol}/L\right)\times 38.665 \end{array}$$

### 2.5 Evaluation of Physical Activity

The International Physical Activity Questionnaires (IPAQ) was used to measure the level of physical activity, consisting of three sections: vigorous activity with a score of 8, moderate activity with a score of 4, and walking with a score of 3.3. The range is 0 to 600 min/week of light activity, 600 to 3000 min of moderate activity, and over 3000 min of vigorous activity. However, an individual must be active for at least 10 min continuously for their score to count [25].

### 2.6 Determination of Sample Size

The sample size was determined based on the study conducted by Chun‐Ming et al. [26] and using the WHtR index with an accuracy (d) of 0.01, a standard deviation (SD) of 0.06, power of 90%, and a confidence level of 95%, which resulted in 400 people.

### 2.7 Statistical Analysis

All quantitative data were described as mean ± standard deviation, and qualitative data as numbers (percentages). An independent *t*‐test was used to compare quantitative variables in two groups (MetS and Non‐MetS), and a Chi‐square test was used to compare qualitative variables. Youden index was used to determine the optimal cut‐off point of each index. The area under the curve (AUC) obtained from the receiver operating characteristic (ROC) curve, along with sensitivity, specificity, and precision, was used to compare different indices. Post hoc power analysis was performed to examine the power of the study in male and female subgroups via MedCalc Software Ltd. Also, the AUC comparison of anthropometric indices had been done with Hanley & McNeil methods in MedCalc software. The odds of metabolic syndrome were evaluated using logistic regression modelling (crude model and models with adjustment of the effect of confounding factors). Metabolic syndrome as the response variable and each of the classified anthropometric criteria (based on the optimal cut‐off point) as the main predictor variable were entered into the logistic regression models. Also, age, gender, race, education, job, duration of disease, physical activity, and medications have been considered as the potential confounding variables in the adjusted models [27, 28, 29, 30, 31]. All analyses were performed in the SPSS software version 26 with a significance level of 5%.

## 3 Results

A total of 400 participants (147 MetS and 253 non‐MetS patients) were involved in the study. The mean ages of the participants in the non‐MetS and MetS groups were 50.86 ± 5.65 and 51.22 ± 5.40 years, respectively. The general characteristics of individuals are shown in Table 1.

**TABLE 1:** The characteristics of the subjects in the non‐MetS and MetS groups.

Variables	Non‐MetS group (*n* = 147)	MetS group (*n* = 253)	*p*
Age (years)	50.86 (5.65)	51.22 (5.40)	0.535
Sex (*N*) (%)			
Male	62 (39.2)	96 (60.8)	0.404*
Female	85 (35.1)	157 (64.9)	
Education (*N*) (%)			
Illiterate‐elementary	87 (39.0)	136 (61.0)	0.584*
Middle‐school	26 (32.5)	54 (67.5)	
High‐school	25 (37.9)	41 (62.1)	
Collage	9 (29.0)	22 (71.0)	
Race (*N*) (%)			
Fars	37 (39.8)	56 (60.2)	0.589*
Lore	57 (33.9)	111 (66.1)	
Arab	53 (38.1)	86 (61.9)	
Job (*N*) (%)			
Unemployed	11 (40.7)	16 (59.3)	0.536*
Labor	26 (33.3)	52 (66.7)	
Housekeeper	83 (39.5)	127 (60.5)	
Employee	27 (31.8)	58 (68.2)	
P.A (met‐min/week)	304.11 (145.90)	301.36 (158.38)	0.863
Duration of disease	7.71 (2.69)	7.45 (2.66)	0.348
SBP (mmHg)	121.81 (8.62)	130.09 (12.12)	< 0.001
DBP (mmHg)	76.46 (6.80)	81.61 (8.43)	< 0.001
MAP (mmHg)	91.58 (6.76)	97.77 (9.00)	< 0.001
PP (mmHg)	45.35 (6.70)	48.48 (8.31)	< 0.001
Traditional anthropometric indices			
Weight (kg)	70.59 (8.65)	77.09 (8.99)	< 0.001
Height (cm)	165.28 (8.65)	165.83 (9.25)	0.554
BMI (kg/m2)	25.88 (2.99)	28.13 (3.30)	< 0.001
WC (cm)	94.57 (7.63)	105.11 (5.35)	< 0.001
HC (cm)	100.42 (6.62)	108.15 (6.26)	< 0.001
WHR	0.94 (0.05)	0.97 (0.05)	< 0.001
Biochemical parameters			
FBG (mg/dL)	152.82 (24.82)	172.13 (36.68)	< 0.001
HbA1c (%)	7.91 (1.03)	8.64 (1.11)	< 0.001
TG (mg/dL)	130.67 (37.79)	169.70 (46.89)	< 0.001
TG (mmol/L)	1.47 (0.42)	1.91 (0.52)	< 0.001
TC (mg/dL)	151.03 (32.56)	164.77 (43.11)	< 0.001
LDL‐c (mg/dL)	81.57 (30.75)	93.33 (39.43)	0.001
HDL‐c (mg/dL)	46.95 (7.83)	40.42 (7.79)	< 0.001
HDL‐c (mmol/L)	1.21 (0.20)	1.04 (0.20)	< 0.001
LDL.HDL‐c	1.78 (0.76)	2.41 (1.24)	< 0.001
Novel anthropometric indices			
WHtR	0.57 (0.06)	0.64 (0.05)	< 0.001
BRI	5.57 (0.54)	6.18 (0.51)	< 0.001
AVI	18.00 (2.93)	22.15 (2.25)	< 0.001
CI	1.33 (0.09)	1.42 (0.09)	< 0.001
WWI	11.29 (0.85)	12.03 (0.89)	< 0.001
TMI	15.74 (2.30)	17.07 (2.63)	< 0.001
BAI	29.50 (4.92)	32.99 (5.67)	< 0.001
VAI	2.21 (0.93)	3.60 (1.37)	< 0.001
RFM	35.77 (7.49)	39.79 (7.50)	< 0.001

*Note:* Values are expressed as mean (SD). *p* < 0.05 was considered as significant using Independent *t*‐test for comparison between the two groups.

Abbreviations: AVI, abdominal volume index; BAI, body adiposity index; BMI, body mass index; BRI, body roundness index; CI, conicity index; DBP, diastolic blood pressure; FBG, fasting blood glucose; HC, hip circumference; HDL‐c, high‐density lipoprotein cholesterol; LDL‐c, low‐density lipoprotein cholesterol; MAP, mean arterial pressure; MetS, metabolic syndrome; PP, pulse pressure; RFM, relative fat mass; SBP, systolic blood pressure; TC, total cholesterol; TG, triglyceride; TMI, triponderal mass index; VAI, visceral adiposity index; WC, waist circumference; WHR, waist‐to‐hip ratio; WHtR, waist‐to‐height ratio; WWI, weight‐adjusted waist index.

* *p* < 0.05 was considered as significant using Chi‐square test.

SBP, DBP, MAP, and PP were significantly higher in the MetS group (*p* < 0.001 for all comparisons). The mean of FBG, HbA1c, TG, TC, HDL‐c, and LDL‐HDL‐c was higher in the MetS group with *p* < 0.001. Also, LDJ‐c was different in the two groups (*p* = 0.001). The mean of weight, BMI, WC, HC, and WHR was greater in the MetS group (*p* < 0.001 for all comparisons). Also, all of the novel anthropometric indices were significantly greater in the MetS group (*p* < 0.001 for all comparisons).

Table 2 shows the optimal cut‐off values for the anthropometric indices based on the total population and each gender, along with the decision criteria. All determined optimal cut‐off values of anthropometric indices had significant AUC in the total population and in different genders (*p* < 0.001). In the total population, AVI had the AUC (0.86). Also, AVI had the highest AUC (0.88) for females and the AUC of VAI was the highest in males (0.88). Although AVI had a higher AUC than VAI and BRI, no statistically significant difference was observed (Table 3). Also, no statistical difference was observed between the AUC of VAI and BRI.

**TABLE 2:** Optimal cut‐off value for anthropometric indices in predicting the risk of MetS in patients with T2DM.

Variable	Cutoff	Sen	Spe	Precision	AUC	AUC 95% CI	Post hoc power	AUC *p*‐value
BMI								
Total	27.29	0.62	0.70	0.78	0.69	(0.64, 0.75)	—	< 0.001
Female	27.85	0.64	0.67	0.78	0.69	(0.62, 0.76)	0.999	< 0.001
Male	26.83	0.54	0.81	0.81	0.69	(0.61, 0.78)	0.986	< 0.001
WHR								
Total	0.97	0.56	0.75	0.79	0.68	(0.63, 0.74)	—	< 0.001
Female	0.92	0.83	0.52	0.76	0.68	(0.60, 0.75)	0.997	< 0.001
Male	0.97	0.64	0.76	0.80	0.71	(0.63, 0.80)	0.997	< 0.001
WHtR								
Total	0.59	0.76	0.74	0.83	0.80	(0.76, 0.85)	—	< 0.001
Female	0.59	0.92	0.67	0.84	0.85	(0.80, 0.91)	> 0.999	< 0.001
Male	0.55	0.90	0.47	0.72	0.75	(0.67, 0.83)	> 0.999	< 0.001
BRI								
Total	5.78	0.76	0.74	0.83	0.81	(0.76, 0.85)	—	< 0.001
Female	5.75	0.92	0.67	0.84	0.85	(0.80, 0.91)	> 0.999	< 0.001
Male	5.59	0.72	0.64	0.76	0.75	(0.68, 0.83)	> 0.999	< 0.001
AVI								
Total	18.82	0.91	0.67	0.83	0.86	(0.82, 0.91)	—	< 0.001
Female	20.00	0.87	0.78	0.88	0.88	(0.82, 0.93)	> 0.999	< 0.001
Male	18.43	0.94	0.65	0.80	0.84	(0.77, 0.91)	> 0.999	< 0.001
Conicity								
Total	1.33	0.87	0.58	0.78	0.76	(0.71, 0.81)	—	< 0.001
Female	1.34	0.89	0.65	0.82	0.79	(0.72, 0.86)	> 0.999	< 0.001
Male	1.33	0.81	0.55	0.74	0.70	(0.62, 0.79)	0.993	< 0.001
WWI								
Total	11.50	0.70	0.69	0.79	0.73	(0.68, 0.78)	—	< 0.001
Female	11.50	0.85	0.66	0.82	0.77	(0.71, 0.84)	> 0.999	< 0.001
Male	10.81	0.90	0.37	0.69	0.64	(0.55, 0.73)	0.847	< 0.001
TMI								
Total	15.81	0.67	0.56	0.72	0.65	(0.59, 0.70)	—	< 0.001
Female	16.99	0.66	0.61	0.76	0.67	(0.60, 0.74)	0.994	< 0.001
Male	15.46	0.51	0.73	0.74	0.62	(0.53, 0.71)	0.726	< 0.001
BAI								
Total	33.90	0.44	0.84	0.83	0.68	(0.62, 0.73)	—	< 0.001
Female	34.12	0.62	0.80	0.85	0.75	(0.68, 0.81)	> 0.999	< 0.001
Male	27.33	0.64	0.58	0.70	0.62	(0.53, 0.71)	0.726	< 0.001
VAI								
Total	2.44	0.85	0.72	0.84	0.85	(0.81, 0.89)	—	< 0.001
Female	2.91	0.82	0.80	0.88	0.85	(0.80, 0.91)	> 0.999	< 0.001
Male	2.36	0.80	0.86	0.90	0.88	(0.82, 0.93)	> 0.999	< 0.001
RFM								
Total	42.21	0.57	0.81	0.84	0.69	(0.64, 0.74)	—	< 0.001
Female	42.21	0.92	0.67	0.84	0.85	(0.80, 0.91)	> 0.999	< 0.001
Male	27.79	0.90	0.47	0.72	0.75	(0.67, 0.83)	> 0.999	< 0.001

Abbreviations: AVI, abdominal volume index; BAI, body adiposity index; BMI, body mass index; BRI, body roundness index; CI, conicity index; RFM, relative fat mass; TMI, triponderal mass index; VAI, visceral adiposity index; WHR, waist‐to‐hip ratio; WHtR, waist‐to‐height ratio; WWI, weight‐adjusted waist index.

**TABLE 3:** Pairwise AUC comparisons of anthropometric indices.

		VAI	BRI	WHtR	Conicity	WWI	BMI	RFM	WHR	BAI	TMI
	AUC	0.85	0.81	0.8	0.76	0.73	0.69	0.69	0.68	0.68	0.65
AVI	0.86	0.699	0.07	0.032	< 0.001	< 0.001	< 0.001	< 0.001	< 0.001	< 0.001	< 0.001
VAI	0.85	—	0.154	0.079	0.003	< 0.001	< 0.001	< 0.001	< 0.001	< 0.001	< 0.001
BRI	0.81		—	0.74	0.113	0.014	< 0.001	< 0.001	< 0.001	< 0.001	< 0.001
WHtR	0.8			—	0.21	0.033	0.001	0.001	< 0.001	< 0.001	< 0.001
Conicity	0.76				—	0.38	0.047	0.024	0.012	0.024	0.002
WWI	0.73					—	0.269	0.269	0.17	0.036	0.031
BMI	0.69						—	1	0.789	0.789	0.293
RFM	0.69							—	0.395	0.789	0.293
WHR	0.68								—	1	0.433
BAI	0.68									—	0.433

Abbreviations: AVI, abdominal volume index; BAI, body adiposity index; BMI, body mass index; BRI, body roundness index; CI, conicity index; RFM, relative fat mass; TMI, triponderal mass index; VAI, visceral adiposity index; WHR, waist‐to‐hip ratio; WHtR, waist‐to‐height ratio; WWI, weight‐adjusted waist index.

The ROC curve of each anthropometric index has been shown in Figures S1–S11 for the total population, males, and females (Figures S1–S11). The AUC of the BMI and VAI was almost the same for both genders (Figures S1 and S10). Except for WHR (Figure S2), the AUC was slightly better in males than in females; all other indices had a better AUC in females.

Table 4 shows the odds ratio (95% CI) of anthropometric indices based on logistic regression. All anthropometric indices increased the odds of MetS significantly with *p* < 0.001 in all crude and adjusted models. AVI had the largest odds ratio for MetS (21.28, 95% CI 12.26–36.92) among all indices in crude models, while the odds ratio of RFM was the highest in model2 (24.52, 95% CI 11.67–51.53) and model 3 (28.2, 95% CI 13.01–61.13).

**TABLE 4:** Odds ratios (95% CI) for MetS according to anthropometric indices.

Variables	*B*	OR (CI)	*p* *	Negelkerke *R* ^2^
BMI				
Model 1^a^	1.34	3.83 (2.48, 5.91)	< 0.001	0.128
Model 2^b^	1.39	4.02 (2.55, 6.32)	< 0.001	0.130
Model 3^c^	1.52	4.55 (2.82, 7.34)	< 0.001	0.185
WHR				
Model 1^a^	1.27	3.54 (2.28, 5.51)	< 0.001	0.111
Model 2^b^	1.29	3.62 (2.32, 5.65)	< 0.001	0.117
Model 3^c^	1.31	3.71 (2.33, 5.89)	< 0.001	0.157
WHtR				
Model 1^a^	2.03	7.62 (4.83, 12.04)	< 0.001	0.261
Model 2^b^	2.28	9.81 (5.85, 16.46)	< 0.001	0.280
Model 3^c^	2.37	10.71 (6.21, 18.47)	< 0.001	0.316
BRI				
Model 1^a^	2.14	8.53 (5.36, 13.58)	< 0.001	0.285
Model 2^b^	2.44	11.45 (6.73, 19.50)	< 0.001	0.307
Model 3^c^	2.54	12.70 (7.24, 22.29)	< 0.001	0.343
AVI				
Model 1^a^	3.06	21.28 (12.26, 36.92)	< 0.001	0.592
Model 2^b^	3.06	21.23 (12.23, 36.86)	< 0.001	0.594
Model 3^c^	3.18	24.06 (13.35, 43.35)	< 0.001	0.626
CI				
Model 1^a^	2.17	8.83 (5.42, 14.38)	< 0.001	0.268
Model 2^b^	2.18	8.87 (5.44, 14.49)	< 0.001	0.270
Model 3^c^	2.32	10.17 (5.98, 17.29)	< 0.001	0.308
WWI				
Model 1^a^	1.63	5.11 (3.29, 7.94)	< 0.001	0.182
Model 2^b^	1.74	5.67 (3.55, 9.05)	< 0.001	0.190
Model 3^c^	1.76	5.79 (3.54, 9.49)	< 0.001	0.219
TMI				
Model 1^a^	0.93	2.54 (1.67, 3.85)	< 0.001	0.065
Model 2^b^	1.00	2.71 (1.72, 4.25)	< 0.001	0.085
Model 3^c^	1.08	2.95 (1.83, 4.76)	< 0.001	0.118
BAI				
Model 1^a^	1.41	4.08 (2.45, 6.80)	< 0.001	0.111
Model 2^b^	1.54	4.65 (2.70, 8.02)	< 0.001	0.118
Model 3^c^	1.54	4.68 (2.68, 8.19)	< 0.001	0.158
VAI				
Model 1^a^	2.65	14.19 (8.64, 23.31)	< 0.001	0.385
Model 2^b^	2.92	18.60 (10.63, 32.54)	< 0.001	0.406
Model 3^c^	3.00	19.98 (11.11, 35.94)	< 0.001	0.430
RFM				
Model 1^a^	1.74	5.71 (3.53, 9.23)	< 0.001	0.187
Model 2^b^	3.20	24.52 (11.67, 51.53)	< 0.001	0.296
Model 3^c^	3.34	28.20 (13.01, 61.13)	< 0.001	0.336

Abbreviations: AVI, abdominal volume index; BAI, body adiposity index; BMI, body mass index; BRI, body roundness index; CI, conicity index; RFM, relative fat mass; TMI, triponderal mass index; VAI, visceral adiposity index; WHR, waist‐to‐hip ratio; WHtR, waist‐to‐height ratio; WWI, weight‐adjusted waist index.

^a^ Model 1: unadjusted.

^b^ Model 2: adjusted for age, gender.

^c^ Model 3: adjustment for age, gender, race, education, job, duration of disease, physical activity, and medications.

* *p* < 0.05 statistically significant by Multivariable logistic regression.

## 4 Discussion

In this cross‐sectional analytical study, we evaluated both traditional and novel anthropometric indices for their ability to predict metabolic syndrome (MetS) in Iranian adults with T2DM. Our findings demonstrated a high prevalence of MetS (63.2%), which is in line with previous reports from Middle Eastern countries and underscores the elevated cardiometabolic burden in this population [7, 32, 33, 34, 35]. The observed prevalence can be attributed to a combination of genetic predisposition, sedentary behaviour, and rising obesity rates, as shown in regional cohort studies [36, 37]. These results emphasise the importance of using anthropometric indices as practical tools for the early identification of MetS among high‐risk diabetic patients.

In this study, both traditional and novel anthropometric indices used in the study were effective in predicting the odds of MetS. A previous study also showed that traditional and novel anthropometric indices were associated with cardiometabolic risk factors in Iranian patients with T2DM [38]. In contrast, in another study, VAI, CI, and LAP did not sufficiently predict CVD risk, and the authors suggested that traditional indices may remain more reliable for Turkish patients with T2DM [39].

Significant differences were observed between MetS and non‐MetS groups across both traditional and novel anthropometric indices. Among these, AVI showed the best overall performance, with the highest sensitivity and AUC, confirming its usefulness for detecting MetS. The VAI also demonstrated strong predictive value, particularly in men, with superior precision and specificity. In women, WHtR, BRI, and RFM had the highest sensitivity, while AVI and VAI remained the strongest overall predictors. The strong performance of AVI and VAI may be explained by their ability to capture visceral fat accumulation, which is known to promote insulin resistance, dyslipidaemia, and hypertension through proinflammatory pathways [40, 41, 42]. Our findings are consistent with recent studies highlighting the superiority of AVI and VAI compared with traditional measures such as BMI [17, 43, 44]. For instance, a recent meta‐analysis in Asian populations confirmed the stronger predictive value of VAI for cardiometabolic outcomes [45], while a longitudinal study in Chinese patients with T2DM demonstrated the long‐term predictive power of AVI for diabetes progression [46]. In women, the high sensitivity of WHtR and BRI also supports earlier reports from Iranian cohorts, where waist‐based indices showed strong associations with central obesity and MetS [47]. By contrast, BMI showed only modest predictive ability, reflecting its known limitations in distinguishing fat from lean mass and ignoring fat distribution [48]. This finding is supported by large international studies that reported lower predictive accuracy of BMI compared with lipid‐ and waist‐based indices [49, 50, 51]. Although the BAI had relatively high specificity, its low sensitivity limits its use as a primary screening tool. Other indices, such as the CI and WWI, also demonstrated predictive value, though to a lesser extent than AVI and VAI. In the present study, gender‐specific analyses provided further insights. Women generally showed higher AUC values, which may reflect the stronger metabolic impact of central obesity in females. On the other hand, WHR performed slightly better in men, likely due to its sensitivity to android fat distribution [52]. Differences in body fat distribution, with a greater tendency toward subcutaneous and gluteofemoral fat accumulation in women and more visceral fat deposition in men, are known to influence cardiometabolic risk [53]. Hormonal factors, including oestrogen deficiency during and after menopause, may further exacerbate central fat accumulation and insulin resistance in women [54]. Behavioural factors such as physical activity patterns and dietary habits may also contribute to these observed sex differences [55]. These observations are in agreement with recent international evidence supporting sex‐related differences in fat distribution and metabolic risk [56, 57, 58]. Findings from a cross‐sectional study indicated that the most predictive anthropometric index for determining MetS differed by sex and across age subgroups in adults with T2DM from Southeast China. BMI showed the largest AUC in men, whereas WHtR and BRI in women [13]. In another cross‐sectional study, the best predictor of MetS was BMI for men and TyG‐WC for women in Chinese adults with T2DM [56].

The optimal cut‐off values identified in this study showed some variation compared with those reported in neighbouring populations. For instance, studies conducted in Chinese adults with T2DM reported lower AVI and VAI thresholds for MetS detection [59, 60], whereas higher cut‐off values have been observed in Turkish populations [16, 61]. Indian studies have also demonstrated population‐specific thresholds, likely reflecting differences in ethnicity, body composition, lifestyle, and cardiometabolic risk profiles [62]. These comparisons highlight the importance of deriving population‐specific cut‐off values rather than extrapolating thresholds across different ethnic groups.

The derived cut‐off points provide clinicians with practical thresholds for risk stratification in patients with T2DM. For example, an AVI threshold of 18.8 and a VAI threshold of 2.4 offered high sensitivity and precision in the overall sample, while sex‐specific values further improved diagnostic accuracy. These indices may be useful not only for screening but also for guiding tailored interventions, such as lifestyle modification in patients with high AVI or lipid‐lowering therapy in those with elevated VAI. The strengths of this study include the relatively large sample size, simultaneous evaluation of multiple indices, and gender‐stratified analyses, all of which enhance its clinical relevance. Furthermore, the use of ROC curves and multivariable regression analyses strengthened the robustness of the findings. However, several limitations should be acknowledged. First, the cross‐sectional design precludes any inference of causal relationships between anthropometric indices and MetS. Second, data were collected from a single centre, which may limit geographic representation and generalisability to the broader Iranian population. Third, physical activity data were self‐reported, introducing the possibility of recall bias. Additionally, dietary intake was not assessed, and direct measures of insulin resistance, such as the homeostasis model assessment of insulin resistance (HOMA‐IR), were not available. Although multivariable analyses were performed, residual confounding due to unmeasured factors cannot be fully excluded. Finally, the sampling method was another limitation of this study. Future longitudinal studies in diverse populations are needed to validate these cut‐off points and to assess their predictive value for long‐term cardiovascular and metabolic outcomes.

## 5 Conclusion

In summary, this study demonstrated that novel anthropometric indices, particularly the AVI and VAI, showed the strongest associations with MetS compared with traditional measures such as BMI in Iranian adults with T2DM. A key contribution of this work is the derivation of Iranian‐specific cut‐off values for these indices, including AVI ≥ 18.8 and VAI ≥ 2.4, which exhibited high diagnostic accuracy. These thresholds may be applied in clinical settings as simple and cost‐effective tools for early risk stratification and screening of MetS in patients with T2DM. Incorporating these indices into routine clinical assessment could improve the early detection of MetS and support timely interventions to reduce cardiometabolic complications. Further longitudinal studies are needed to confirm their predictive validity across diverse populations.

## Author Contributions

**Vahideh Aghamohammadi:** data curation, methodology, investigation, writing – original draft, writing – review and editing, validation. **Ahmad Zare Javid:** writing – review and editing, validation, methodology, data curation. **Hadi Bazyar:** conceptualization, investigation, methodology, validation, formal analysis, data curation, funding acquisition, supervision. **Mohammad Reza Amini:** methodology, validation, writing – review and editing, data curation. **Mohammad Moqaddasi Amiri:** data curation, resources, writing – original draft, writing – review and editing, formal analysis, methodology, investigation, validation.

## Funding

This work has been financially supported by the Sirjan School of Medical Sciences.

## Conflicts of Interest

The authors declare no conflicts of interest.

## Supporting information


**Figure S1:** Roc Curve for BMI (A. total with AUC = 0.69 (95% CI: 0.64–0.75), B. female with AUC = 0.69 (95% CI: 0.62–0.76) and male with AUC = 0.69 (95% CI: 0.61–0.78)).
**Figure S2:** Roc Curve for WHR (A. total with AUC = 0.68 (95% CI: 0.63–0.74), B. female with AUC = 0.68 (95% CI: 0.60–0.75) and male with AUC = 0.71 (95% CI: 0.63–0.80)).
**Figure S3:** Roc Curve for WHtR (A. total with AUC = 0.80 (95% CI: 0.76–0.85), B. female with AUC = 0.85 (95% CI: 0.80–0.91) and male with AUC = 0.75 (95% CI: 0.67–0.83)).
**Figure S4:** Roc Curve for BRI (A. total with AUC = 0.81 (95% CI: 0.76–0.85), B. female with AUC = 0.85 (95% CI: 0.80–0.91) and male with AUC = 0.75 (95% CI: 0.68–0.83)).
**Figure S5:** Roc Curve for AVI (A. total with AUC = 0.86 (95% CI: 0.82–0.91), B. female with AUC = 0.88 (95% CI: 0.82–0.93) and male with AUC = 0.84 (95% CI: 0.77–0.91)).
**Figure S6:** Roc Curve for Conicity (A. total with AUC = 0.76 (95% CI: 0.71–0.81), B. female with AUC = 0.79 (95% CI: 0.72–0.86) and male with AUC = 0.70 (95% CI: 0.62–0.79)).
**Figure S7:** Roc Curve for WWI (A. total with AUC = 0.73 (95% CI: 0.68–0.78), B. female with AUC = 0.77 (95% CI: 0.71–0.84) and male with AUC = 0.64 (95% CI: 0.55–0.73)).
**Figure S8:** Roc Curve for TMI (A. total with AUC = 0.65 (95% CI: 0.59–0.70), B. female with AUC = 0.67 (95% CI: 0.60–0.74) and male with AUC = 0.62 (95% CI: 0.53–0.71)).
**Figure S9:** Roc Curve for BAI (A. total with AUC = 0.68 (95% CI: 0.62–0.73), B. female with AUC = 0.75 (95% CI: 0.68–0.81) and male with AUC = 0.62 (95% CI: 0.53–0.71)).
**Figure S10:** Roc Curve for VAI (A. total with AUC = 0.85 (95% CI: 0.81–0.89), B. female with AUC = 0.85 (95% CI: 0.80–0.91) and male with AUC = 0.88 (95% CI: 0.82–0.93)).
**Figure S11:** Roc Curve for RFM (A. total with AUC = 0.69 (95% CI: 0.64–0.74), B. female with AUC = 0.85 (95% CI: 0.80–0.91) and male with AUC = 0.75 (95% CI: 0.67–0.83)).

## Data Availability

The datasets used and/or analysed during the current study are available from the corresponding author on reasonable request.

## References

[1] K. G. Alberti , R. H. Eckel , S. M. Grundy , et al., “Harmonizing the Metabolic Syndrome: A Joint Interim Statement of the International Diabetes Federation Task Force on Epidemiology and Prevention; National Heart, Lung, and Blood Institute; American Heart Association; World Heart Federation; International Atherosclerosis Society; and International Association for the Study of Obesity,” Circulation 120, no. 16 (2009): 1640–1645. 19805654 10.1161/CIRCULATIONAHA.109.192644

[2] M.‐A. Cornier , D. Dabelea , T. L. Hernandez , et al., “The Metabolic Syndrome,” Endocrine Reviews 29, no. 7 (2008): 777–822. 18971485 10.1210/er.2008-0024 PMC5393149

[3] O. Tabatabaei‐Malazy , S. Saeedi Moghaddam , N. Rezaei , et al., “A Nationwide Study of Metabolic Syndrome Prevalence in Iran; a Comparative Analysis of Six Definitions,” PLoS One 16, no. 3 (2021): e0241926. 33657130 10.1371/journal.pone.0241926 PMC7928520

[4] H. Sun , P. Saeedi , S. Karuranga , et al., “IDF Diabetes Atlas: Global, Regional and Country‐Level Diabetes Prevalence Estimates for 2021 and Projections for 2045,” Diabetes Research and Clinical Practice 183 (2022): 109119. 34879977 10.1016/j.diabres.2021.109119 PMC11057359

[5] A. Esteghamati , B. Larijani , M. H. Aghajani , et al., “Diabetes in Iran: Prospective Analysis From First Nationwide Diabetes Report of National Program for Prevention and Control of Diabetes (NPPCD‐2016),” Scientific Reports 7, no. 1 (2017): 13461. 29044139 10.1038/s41598-017-13379-z PMC5647418

[6] M. Mirzaei , M. Rahmaninan , M. Mirzaei , A. Nadjarzadeh , and A. A. Dehghani Tafti , “Epidemiology of Diabetes Mellitus, Pre‐Diabetes, Undiagnosed and Uncontrolled Diabetes in Central Iran: Results From Yazd Health Study,” BMC Public Health 20, no. 1 (2020): 166. 32013917 10.1186/s12889-020-8267-y PMC6998152

[7] Z. Foroozanfar , H. Najafipour , N. Khanjani , A. Bahrampour , and H. Ebrahimi , “The Prevalence of Metabolic Syndrome According to Different Criteria and Its Associated Factors in Type 2 Diabetic Patients in Kerman, Iran,” Iranian Journal of Medical Sciences 40, no. 6 (2015): 522. 26538781 PMC4628143

[8] M. Janghorbani and M. Amini , “Metabolic Syndrome in Type 2 Diabetes Mellitus in Isfahan, Iran: Prevalence and Risk Factors,” Metabolic Syndrome and Related Disorders 5, no. 3 (2007): 243–254. 18370778 10.1089/met.2005.0010

[9] M. Hanefeld , C. Koehler , S. Gallo , I. Benke , and P. Ott , “Impact of the Individual Components of the Metabolic Syndrome and Their Different Combinations on the Prevalence of Atherosclerotic Vascular Disease in Type 2 Diabetes: The Diabetes in Germany (DIG) Study,” Cardiovascular Diabetology 6, no. 1 (2007): 13. 17462080 10.1186/1475-2840-6-13 PMC1871572

[10] M.‐F. Yao , J. He , X. Sun , et al., “Gender Differences in Risks of Coronary Heart Disease and Stroke in Patients With Type 2 Diabetes Mellitus and Their Association With Metabolic Syndrome in China,” International Journal of Endocrinology 2016, no. 1 (2016): 8483405. 28042294 10.1155/2016/8483405 PMC5155098

[11] S. Zhu , L. A. McClure , H. Lau , et al., “Recurrent Vascular Events in Lacunar Stroke Patients With Metabolic Syndrome and/or Diabetes,” Neurology 85, no. 11 (2015): 935–941. 26296518 10.1212/WNL.0000000000001933 PMC4567462

[12] C. K. Endukuru , G. S. Gaur , Y. Dhanalakshmi , J. Sahoo , and B. Vairappan , “Cut‐Off Values and Clinical Efficacy of Body Roundness Index and Other Novel Anthropometric Indices in Identifying Metabolic Syndrome and Its Components Among Southern‐Indian Adults,” Diabetology International 13, no. 1 (2022): 188–200. 35059255 10.1007/s13340-021-00522-5 PMC8733072

[13] X. Guo , Q. Ding , and M. Liang , “Evaluation of Eight Anthropometric Indices for Identification of Metabolic Syndrome in Adults With Diabetes,” Diabetes, Metabolic Syndrome and Obesity 14 (2021): 1431–1443. 10.2147/DMSO.S294244 PMC8019619 33833536

[14] S. Khosravian , M. A. Bayani , S. R. Hosseini , A. Bijani , S. Mouodi , and R. Ghadimi , “Comparison of Anthropometric Indices for Predicting the Risk of Metabolic Syndrome in Older Adults,” Romanian Journal of Internal Medicine 59, no. 1 (2021): 43–49. 32881711 10.2478/rjim-2020-0026

[15] H. Wang , A. Liu , T. Zhao , et al., “Comparison of Anthropometric Indices for Predicting the Risk of Metabolic Syndrome and Its Components in Chinese Adults: A Prospective, Longitudinal Study,” BMJ Open 7, no. 9 (2017): e016062. 10.1136/bmjopen-2017-016062 PMC5623484 28928179

[16] E. Ozturk and H. Yildiz , “Evaluation of Different Anthropometric Indices for Predicting Metabolic Syndrome,” European Review for Medical and Pharmacological Sciences 26, no. 22 (2022): 8317–8325. 36459015 10.26355/eurrev_202211_30364

[17] L. Quaye , W. K. B. A. Owiredu , N. Amidu , P. P. M. Dapare , and Y. Adams , “Comparative Abilities of Body Mass Index, Waist Circumference, Abdominal Volume Index, Body Adiposity Index, and Conicity Index as Predictive Screening Tools for Metabolic Syndrome Among Apparently Healthy Ghanaian Adults,” Journal of Obesity 2019, no. 1 (2019): 8143179. 31565431 10.1155/2019/8143179 PMC6745169

[18] S. Głuszek , E. Ciesla , M. Głuszek‐Osuch , et al., “Anthropometric Indices and Cut‐Off Points in the Diagnosis of Metabolic Disorders,” PLoS One 15, no. 6 (2020): e0235121. 32569336 10.1371/journal.pone.0235121 PMC7307766

[19] M. D. DeBoer , “Ethnicity, Obesity and the Metabolic Syndrome: Implications on Assessing Risk and Targeting Intervention,” Expert Review of Endocrinology & Metabolism 6, no. 2 (2011): 279–289. 21643518 10.1586/eem.11.17 PMC3105461

[20] S. A. Lear and D. Gasevic , “Ethnicity and Metabolic Syndrome: Implications for Assessment, Management and Prevention,” Nutrients 12, no. 1 (2019): 15. 31861719 10.3390/nu12010015 PMC7019432

[21] P. Amiri , A. Z. Javid , L. Moradi , et al., “Associations Between New and Old Anthropometric Indices With Type 2 Diabetes Mellitus and Risk of Metabolic Complications: A Cross‐Sectional Analytical Study,” Jornal Vascular Brasileiro 20 (2021): e20200236. 34630540 10.1590/1677-5449.200236 PMC8483943

[22] F. Azizi , D. Khalili , H. Aghajani , et al., “Appropriate Waist Circumference Cut‐Off Points Among Iranian Adults: The First Report of the Iranian National Committee of Obesity,” Archives of Iranian Medicine Journal 13, no. 3 (2010): 243–244. 20433230

[23] H. Bazyar , A. Zare Javid , A. Ahangarpour , et al., “The Effects of Rutin Supplement on Blood Pressure Markers, Some Serum Antioxidant Enzymes, and Quality of Life in Patients With Type 2 Diabetes Mellitus Compared With Placebo,” Frontiers in Nutrition 10 (2023): 1214420. 37599700 10.3389/fnut.2023.1214420 PMC10435270

[24] E. Ehrampoush , P. Arasteh , R. Homayounfar , et al., “New Anthropometric Indices or Old Ones: Which Is the Better Predictor of Body Fat?,” Diabetes and Metabolic Syndrome: Clinical Research and Reviews 11, no. 4 (2017): 257–263. 10.1016/j.dsx.2016.08.027 27578617

[25] C. L. Craig , A. L. Marshall , M. Sjöström , et al., “International Physical Activity Questionnaire: 12‐Country Reliability and Validity,” Medicine & Science in Sports & Exercise 35, no. 8 (2003): 1381–1395. 12900694 10.1249/01.MSS.0000078924.61453.FB

[26] X. Zhang , T. Zhang , S. He , et al., “RETRACTED ARTICLE: Association of Metabolic Syndrome With TyG Index and TyG‐Related Parameters in an Urban Chinese Population: A 15‐Year Prospective Study,” Diabetology & Metabolic Syndrome 14, no. 1 (2022): 84. 35706038 10.1186/s13098-022-00855-4 PMC9202163

[27] Y. Pan and C. A. Pratt , “Metabolic Syndrome and Its Association With Diet and Physical Activity in US Adolescents,” Journal of the American Dietetic Association 108, no. 2 (2008): 276–286. 18237576 10.1016/j.jada.2007.10.049

[28] L. Razzouk and P. Muntner , “Ethnic, Gender, and Age‐Related Differences in Patients With the Metabolic Syndrome,” Current Hypertension Reports 11, no. 2 (2009): 127–132. 19278602 10.1007/s11906-009-0023-8

[29] C. R. Stephens , J. F. Easton , A. Robles‐Cabrera , et al., “The Impact of Education and Age on Metabolic Disorders,” Frontiers in Public Health 8 (2020): 180. 32671006 10.3389/fpubh.2020.00180 PMC7326131

[30] B. Strasser , “Physical Activity in Obesity and Metabolic Syndrome,” Annals of the New York Academy of Sciences 1281, no. 1 (2013): 141–159. 23167451 10.1111/j.1749-6632.2012.06785.x PMC3715111

[31] M. Strauss , C. J. Lavie , G. Lippi , et al., “A Systematic Review of Prevalence of Metabolic Syndrome in Occupational Groups–Does Occupation Matter in the Global Epidemic of Metabolic Syndrome?,” Progress in Cardiovascular Diseases 75 (2022): 69–77. 36162483 10.1016/j.pcad.2022.09.003

[32] R. Belete , Z. Ataro , A. Abdu , and M. Sheleme , “Global Prevalence of Metabolic Syndrome Among Patients With Type I Diabetes Mellitus: A Systematic Review and Meta‐Analysis,” Diabetology & Metabolic Syndrome 13, no. 1 (2021): 25. 33653388 10.1186/s13098-021-00641-8 PMC7923483

[33] İ. Dündar and A. Akıncı , “Prevalence of Type 2 Diabetes Mellitus, Metabolic Syndrome, and Related Morbidities in Overweight and Obese Children,” Journal of Pediatric Endocrinology and Metabolism 35, no. 4 (2022): 435–441. 35026882 10.1515/jpem-2021-0271

[34] A. Bahar , Z. Kashi , M. Kheradmand , et al., “Prevalence of Metabolic Syndrome Using International Diabetes Federation, National Cholesterol Education Panel‐Adult Treatment Panel III and Iranian Criteria: Results of Tabari Cohort Study,” Journal of Diabetes & Metabolic Disorders 19, no. 1 (2020): 205–211. 32550169 10.1007/s40200-020-00492-6 PMC7270474

[35] A. Kanaya , C. Wassel , D. Mathur , et al., “Prevalence and Correlates of Diabetes in South Asian Indians in the United States: Findings From the Metabolic Syndrome and Atherosclerosis in South Asians Living in America Study and the Multi‐Ethnic Study of Atherosclerosis,” Metabolic Syndrome and Related Disorders 8, no. 2 (2010): 157–164. 19943798 10.1089/met.2009.0062 PMC3139526

[36] S. Asghar , S. Asghar , S. Shahid , M. Fatima , S. M. H. Bukhari , and S. N. Siddiqui , “Metabolic Syndrome in Type 2 Diabetes Mellitus Patients: Prevalence, Risk Factors, and Associated Microvascular Complications,” Cureus 15, no. 5 (2023): e39076. 37323312 10.7759/cureus.39076 PMC10268561

[37] A. Riaz , S. Asghar , S. Shahid , H. Tanvir , M. H. Ejaz , and M. Akram , “Prevalence of Metabolic Syndrome and Its Risk Factors Influence on Microvascular Complications in Patients With Type 1 and Type 2 Diabetes Mellitus,” Cureus 16, no. 3 (2024): e55478. 38571859 10.7759/cureus.55478 PMC10989210

[38] S. Golabi , S. Ajloo , F. Maghsoudi , M. Adelipour , and M. Naghashpour , “Associations Between Traditional and Non‐Traditional Anthropometric Indices and Cardiometabolic Risk Factors Among Inpatients With Type 2 Diabetes Mellitus: A Cross‐Sectional Study,” Journal of International Medical Research 49, no. 10 (2021): 03000605211049960. 34657502 10.1177/03000605211049960 PMC8524710

[39] S. Arslan , K. Sahin , N. Dal , R. M. Atan , and K. T. Selcuk , “The Relationship Between Anthropometric Indices and Cardiovascular Risk in Patients With Type 2 Diabetes Mellitus,” Diabetes Research and Clinical Practice 224 (2025): 112243. 40349849 10.1016/j.diabres.2025.112243

[40] H. Kolb , “Obese Visceral Fat Tissue Inflammation: From Protective to Detrimental?,” BMC Medicine 20, no. 1 (2022): 494. 36575472 10.1186/s12916-022-02672-y PMC9795790

[41] N. E. Antonio‐Villa , O. Y. Bello‐Chavolla , A. Vargas‐Vázquez , et al., “Increased Visceral Fat Accumulation Modifies the Effect of Insulin Resistance on Arterial Stiffness and Hypertension Risk,” Nutrition, Metabolism, and Cardiovascular Diseases 31, no. 2 (2021): 506–517. 10.1016/j.numecd.2020.09.031 33279372

[42] H. F. Lopes , M. L. Corrêa‐Giannella , F. M. Consolim‐Colombo , and B. M. Egan , “Visceral Adiposity Syndrome,” Diabetology & Metabolic Syndrome 8, no. 1 (2016): 40. 27437032 10.1186/s13098-016-0156-2 PMC4950710

[43] M.‐T. Tsou , Y.‐C. Chang , C.‐P. Hsu , et al., “Visceral Adiposity Index Outperforms Conventional Anthropometric Assessments as Predictor of Diabetes Mellitus in Elderly Chinese: A Population‐Based Study,” Nutrition & Metabolism 18, no. 1 (2021): 87. 34563209 10.1186/s12986-021-00608-6 PMC8465784

[44] Y. Duan , W. Zhang , Z. Li , et al., “Predictive Ability of Obesity‐ and Lipid‐Related Indicators for Metabolic Syndrome in Relatively Healthy Chinese Adults,” Frontiers in Endocrinology 13 (2022): 1016581. 36465613 10.3389/fendo.2022.1016581 PMC9715593

[45] F. Shen , C. Guo , D. Zhang , Y. Liu , and P. Zhang , “Visceral Adiposity Index as a Predictor of Type 2 Diabetes Mellitus Risk: A Systematic Review and Dose–Response Meta‐Analysis,” Nutrition, Metabolism, and Cardiovascular Diseases 34, no. 4 (2024): 811–822. 10.1016/j.numecd.2023.04.009 38326187

[46] X. Hu , A. A. Appleton , Y. Ou , et al., “Abdominal Volume Index Trajectories and Risk of Diabetes Mellitus: Results From the China Health and Nutrition Survey,” Journal of Diabetes Investigation 13, no. 5 (2022): 868–877. 34902230 10.1111/jdi.13733 PMC9077741

[47] A. Rahimi , S. Rafati , A. Azarbad , et al., “The Predictive Power of Conventional and Novel Obesity Indices in Identifying Metabolic Syndrome Among the Southern Iranian Populations: Findings From PERSIAN Cohort Study,” Journal of Health, Population and Nutrition 43, no. 1 (2024): 198. 39609899 10.1186/s41043-024-00703-3 PMC11606048

[48] A. Bener , M. T. Yousafzai , S. Darwish , A. O. Al‐Hamaq , E. A. Nasralla , and M. Abdul‐Ghani , “Obesity Index That Better Predict Metabolic Syndrome: Body Mass Index, Waist Circumference, Waist Hip Ratio, or Waist Height Ratio,” Journal of Obesity 2013, no. 1 (2013): 269038. 24000310 10.1155/2013/269038 PMC3755383

[49] R. Ajeen , K. I. Turk‐Adawi , A. S. Ammerman , J. A. Batsis , S. W. Ng , and L. S. Adair , “Assessing the Relationship Between Anthropometric Indices and Visceral Adipose Tissue: A Cross‐Sectional Study in a Qatari Population,” Journal of International Medical Research 53, no. 9 (2025): 03000605251371603. 40907031 10.1177/03000605251371603 PMC12411730

[50] C. Huang , A. Lopes , and A. Britton , “Which Adiposity Index Is Best? Comparison of Five Indicators and Their Ability to Identify Type 2 Diabetes Risk in a Population Study,” Diabetes Research and Clinical Practice 225 (2025): 112268. 40404050 10.1016/j.diabres.2025.112268 PMC13408304

[51] S. A. Patel , M. Deepa , R. Shivashankar , et al., “Comparison of Multiple Obesity Indices for Cardiovascular Disease Risk Classification in South Asian Adults: The CARRS Study,” PLoS One 12, no. 4 (2017): e0174251. 28448582 10.1371/journal.pone.0174251 PMC5407781

[52] L. Liu , Y. Liu , X. Sun , et al., “Identification of an Obesity Index for Predicting Metabolic Syndrome by Gender: The Rural Chinese Cohort Study,” BMC Endocrine Disorders 18, no. 1 (2018): 54. 30081888 10.1186/s12902-018-0281-z PMC6090693

[53] S. Sam , “Differential Effect of Subcutaneous Abdominal and Visceral Adipose Tissue on Cardiometabolic Risk,” Hormone Molecular Biology and Clinical Investigation 33, no. 1 (2018): 20180014. 10.1515/hmbci-2018-0014 29522417

[54] J. Zhu , Y. Zhou , B. Jin , and J. Shu , “Role of Estrogen in the Regulation of Central and Peripheral Energy Homeostasis: From a Menopausal Perspective,” Therapeutic Advances in Endocrinology and Metabolism 14 (2023): 20420188231199359. 37719789 10.1177/20420188231199359 PMC10504839

[55] M. Lombardo , A. Feraco , A. Armani , et al., “Gender Differences in Body Composition, Dietary Patterns, and Physical Activity: Insights From a Cross‐Sectional Study,” Frontiers in Nutrition 11 (2024): 1414217. 39055386 10.3389/fnut.2024.1414217 PMC11271261

[56] J. Mao , S. Gan , Q. Zhou , H. Zhou , and Z. Deng , “Optimal Anthropometric Indicators and Cut Points for Predicting Metabolic Syndrome in Chinese Patients With Type 2 Diabetes Mellitus by Gender,” Diabetes, Metabolic Syndrome and Obesity 16 (2023): 505–514. 10.2147/DMSO.S399275 PMC9961221 36852179

[57] A. Kuerban , “Beyond Asian‐Specific Cutoffs: Gender Effects on the Predictability of Body Mass Index, Waist Circumference, and Waist Circumference to Height Ratio on Hemoglobin A1c,” Journal of Racial and Ethnic Health Disparities 8, no. 2 (2021): 415–421. 32542494 10.1007/s40615-020-00796-6

[58] A. Mungvongsa , S. Potivichayanon , C. Dechsukhum , T. Somdee , and S. Paenngoen , “Efficacy of Anthropometric Indicators as Risk Predictors of Metabolic Syndrome Among Academic Staff: A Gender‐Specific, Cross‐Sectional Study,” Journal of the Medical Association of Thailand 108, no. 4 (2025): 321–329.

[59] M. C. Amato and C. Giordano , “Visceral Adiposity Index: An Indicator of Adipose Tissue Dysfunction,” International Journal of Endocrinology 2014, no. 1 (2014): 730827. 24829577 10.1155/2014/730827 PMC4009335

[60] M. Tang , X.‐H. Wei , H. Cao , et al., “Association Between Chinese Visceral Adiposity Index and Metabolic‐Associated Fatty Liver Disease in Chinese Adults With Type 2 Diabetes Mellitus,” Frontiers in Endocrinology 13 (2022): 935980. 35979441 10.3389/fendo.2022.935980 PMC9376620

[61] I. Baloglu , K. Turkmen , N. Y. Selcuk , et al., “The Relationship Between Visceral Adiposity Index and Epicardial Adipose Tissue in Patients With Type 2 Diabetes Mellitus,” Experimental and Clinical Endocrinology & Diabetes 129, no. 5 (2021): 390–395. 31060096 10.1055/a-0892-4290

[62] K. S. Jog , S. Eagappan , R. K. Santharam , S. Subbiah , and R. Santharam , “Comparison of Novel Biomarkers of Insulin Resistance With Homeostasis Model Assessment of Insulin Resistance, Its Correlation to Metabolic Syndrome in South Indian Population and Proposition of Population Specific Cutoffs for These Indices,” Cureus 15, no. 1 (2023): e33653. 36788883 10.7759/cureus.33653 PMC9915858

