# Supplementary material for: Determination of the Optimal Cut‐Off Point of Anthropometric Indices to Predict the Risk of Metabolic Syndrome in Iranian Adult Population With Type 2 Diabetes Mellitus: A Cross‐Sectional‐Analytical Study
Source: Endocrinol Diabetes Metab. 2026 May 15;9(3):e70231. doi: 10.1002/edm2.70231 (PMC13176952; doi:10.1002/edm2.70231)
Supplement: Supplementary file 1 — Figure S1: Roc Curve for BMI (A. total with AUC = 0.69 (95% CI: 0.64–0.75), B. female with AUC = 0.69 (95% CI: 0.62–0.76) and male with AUC = 0.69 (95% CI: 0.61–0.78)). Figure S2: Roc Curve for WHR (A. total with AUC = 0.68 (95% CI: 0.63–0.74), B. female with AUC = 0.68 (95% CI: 0.60–0.75) and male with AUC = 0.71 (95% CI: 0.63–0.80)). Figure S3: Roc Curve for WHtR (A. total with AUC = 0.80 (95% CI: 0.76–0.85), B. female with AUC = 0.85 (95% CI: 0.80–0.91) and male with AUC = 0.75 (95% CI: 0.67–0.83)). Figure S4: Roc Curve for BRI (A. total with AUC = 0.81 (95% CI: 0.76–0.85), B. female with AUC = 0.85 (95% CI: 0.80–0.91) and male with AUC = 0.75 (95% CI: 0.68–0.83)). Figure S5: Roc Curve for AVI (A. total with AUC = 0.86 (95% CI: 0.82–0.91), B. female with AUC = 0.88 (95% CI: 0.82–0.93) and male with AUC = 0.84 (95% CI: 0.77–0.91)). Figure S6: Roc Curve for Conicity (A. total with AUC = 0.76 (95% CI: 0.71–0.81), B. female with AUC = 0.79 (95% CI: 0.72–0.86) and male with AUC = 0.70 (95% CI: 0.62–0.79)). Figure S7: Roc Curve for WWI (A. total with AUC = 0.73 (95% CI: 0.68–0.78), B. female with AUC = 0.77 (95% CI: 0.71–0.84) and male with AUC = 0.64 (95% CI: 0.55–0.73)). Figure S8: Roc Curve for TMI (A. total with AUC = 0.65 (95% CI: 0.59–0.70), B. female with AUC = 0.67 (95% CI: 0.60–0.74) and male with AUC = 0.62 (95% CI: 0.53–0.71)). Figure S9: Roc Curve for BAI (A. total with AUC = 0.68 (95% CI: 0.62–0.73), B. female with AUC = 0.75 (95% CI: 0.68–0.81) and male with AUC = 0.62 (95% CI: 0.53–0.71)). Figure S10: Roc Curve for VAI (A. total with AUC = 0.85 (95% CI: 0.81–0.89), B. female with AUC = 0.85 (95% CI: 0.80–0.91) and male with AUC = 0.88 (95% CI: 0.82–0.93)). Figure S11: Roc Curve for RFM (A. total with AUC = 0.69 (95% CI: 0.64–0.74), B. female with AUC = 0.85 (95% CI: 0.80–0.91) and male with AUC = 0.75 (95% CI: 0.67–0.83)). [file EDM2-9-e70231-s001.docx]

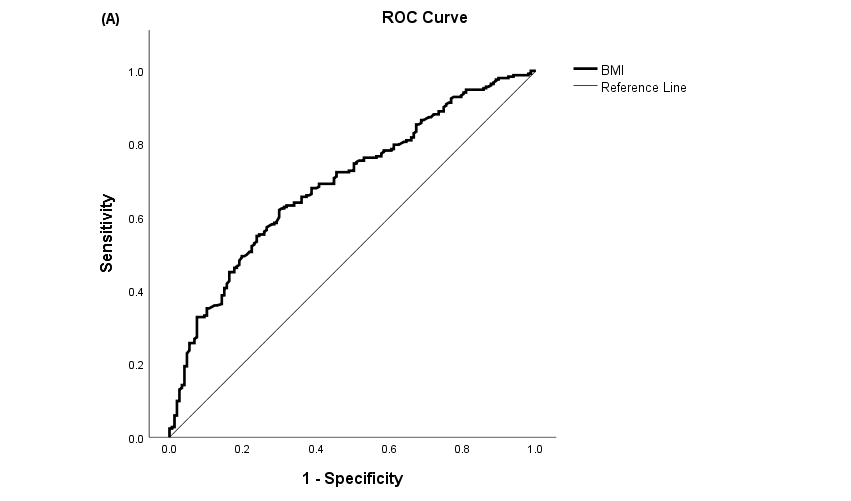


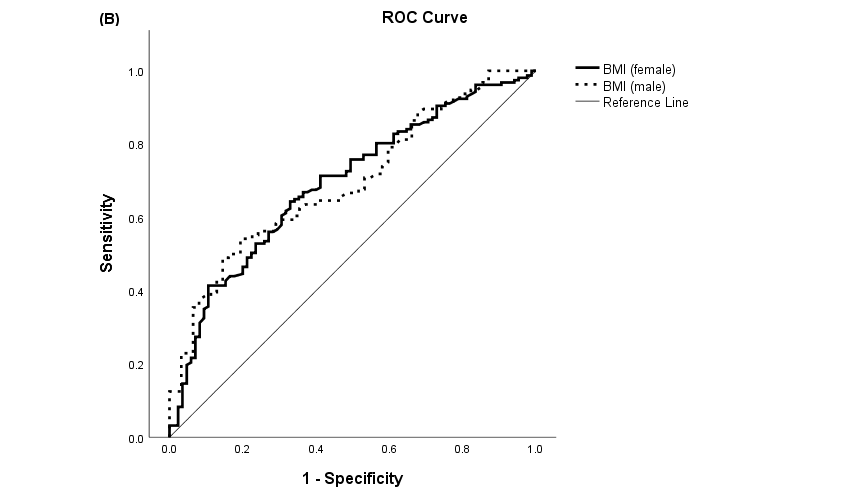


Figure S1: Roc Curve for BMI (A. total with AUC=0.69 (95% CI: 0.64-0.75), B. female with AUC=0.69 (95% CI: 0.62-0.76) and male with AUC=0.69 (95% CI: 0.61-0.78))


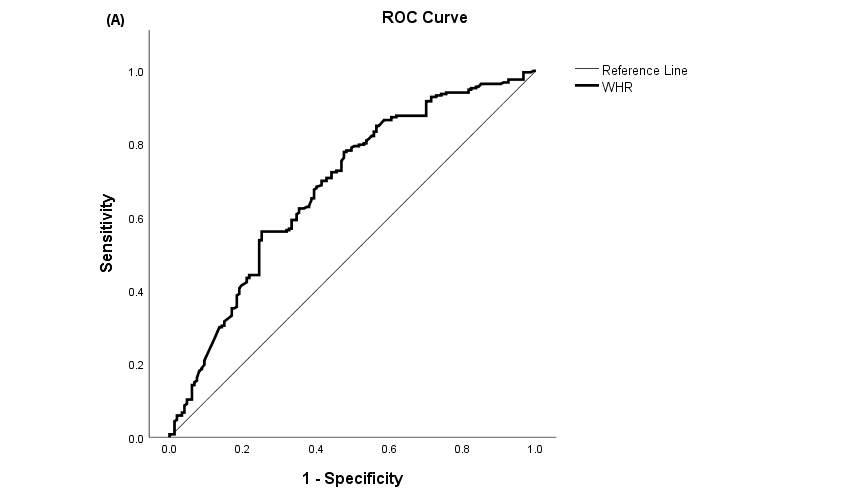


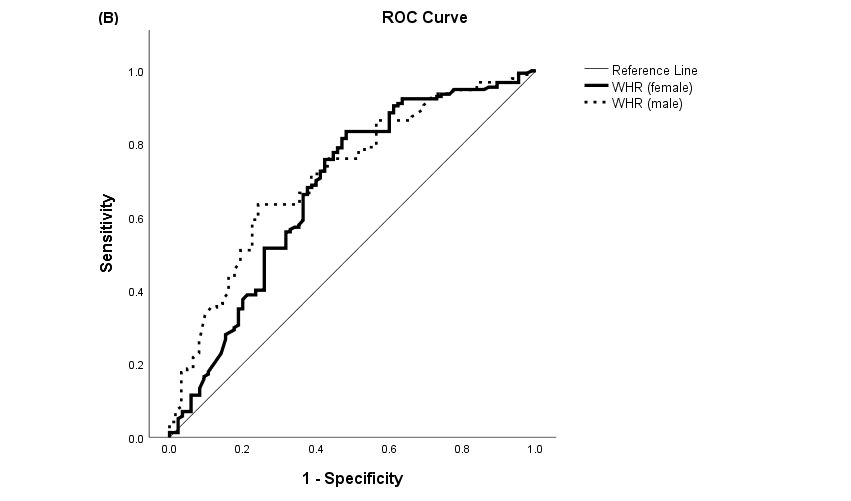


Figure S2: Roc Curve for WHR (A. total with AUC=0.68 (95% CI: 0.63-0.74), B. female with AUC=0.68 (95% CI: 0.60-0.75) and male with AUC=0.71 (95% CI: 0.63-0.80))


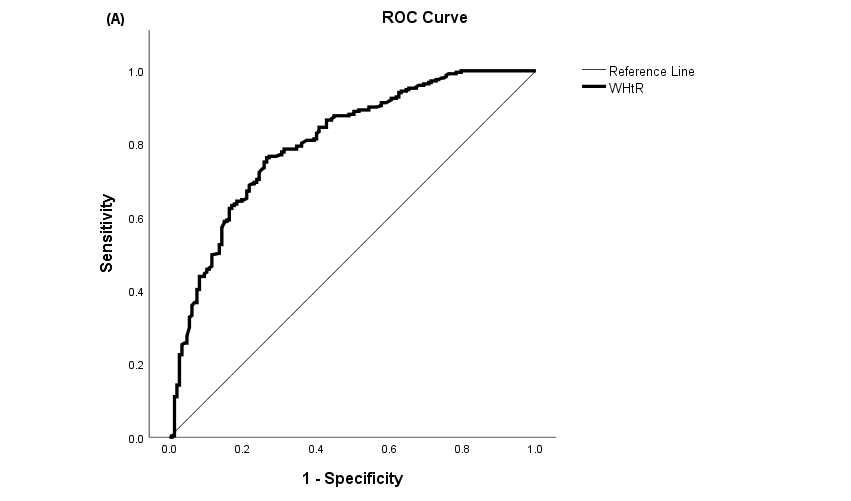


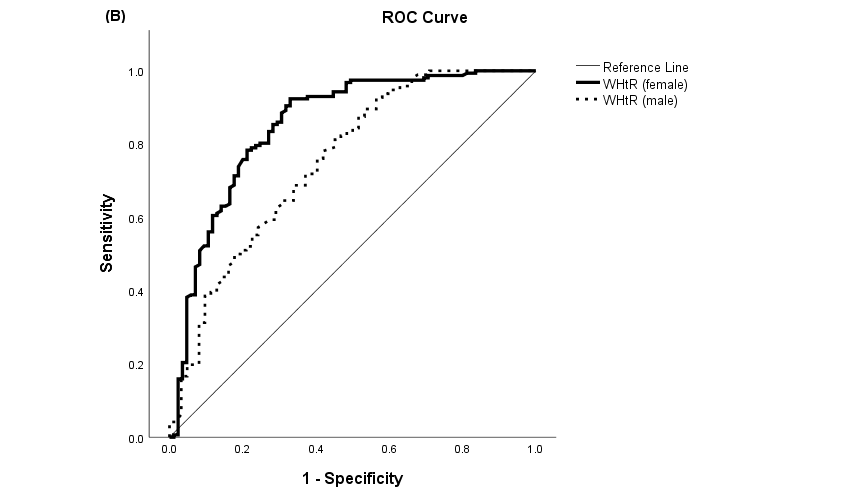


Figure S3: Roc Curve for WHtR (A. total with AUC=0.80 (95% CI: 0.76-0.85), B. female with AUC=0.85 (95% CI: 0.80-0.91) and male with AUC=0.75 (95% CI: 0.67-0.83))


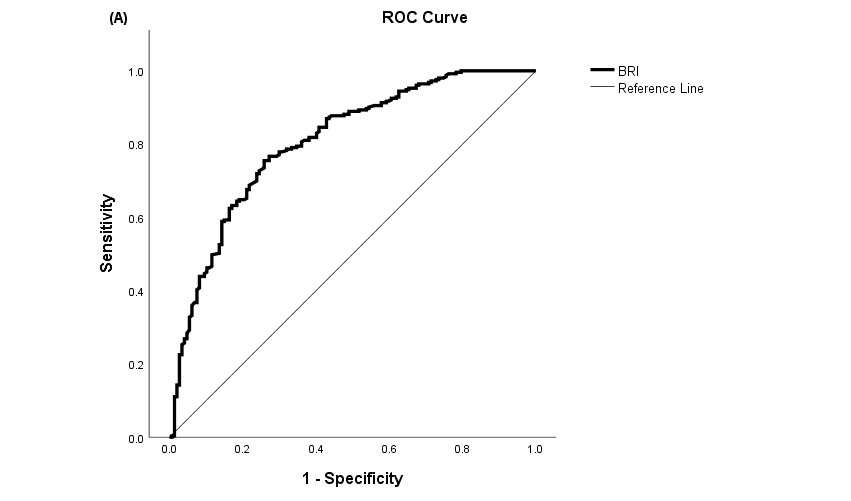


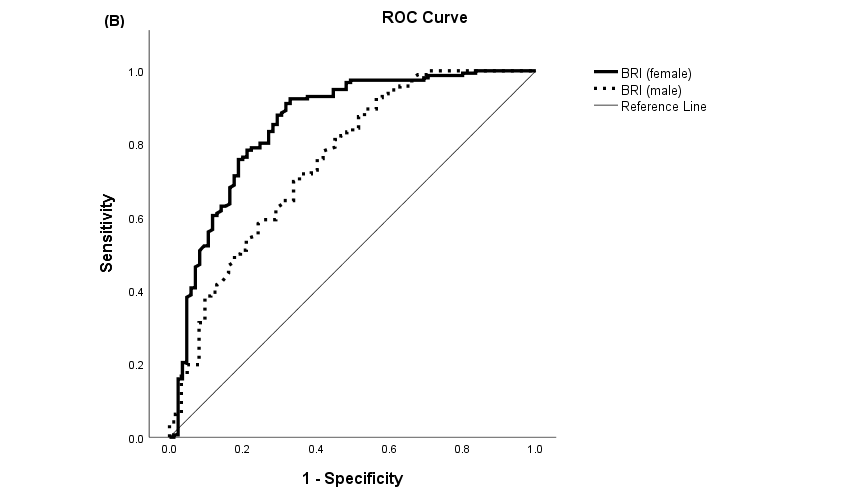


Figure S4: Roc Curve for BRI (A. total with AUC=0.81 (95% CI: 0.76-0.85), B. female with AUC=0.85 (95% CI: 0.80-0.91) and male with AUC=0.75 (95% CI: 0.68-0.83))


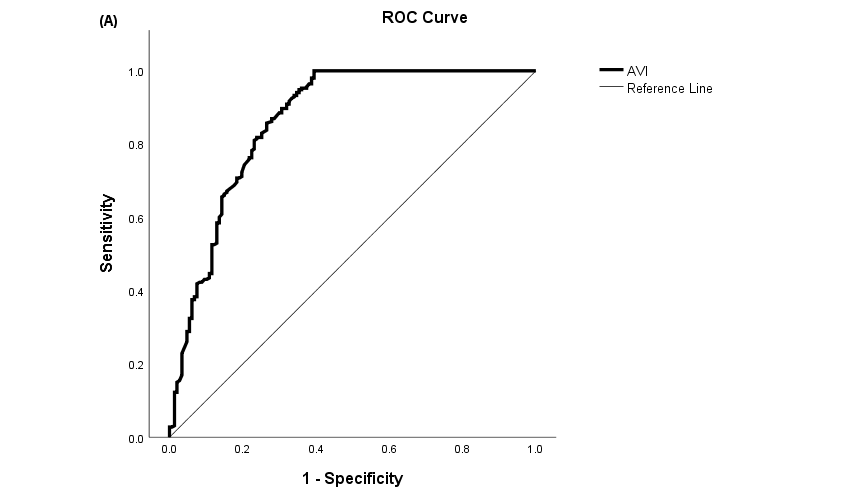


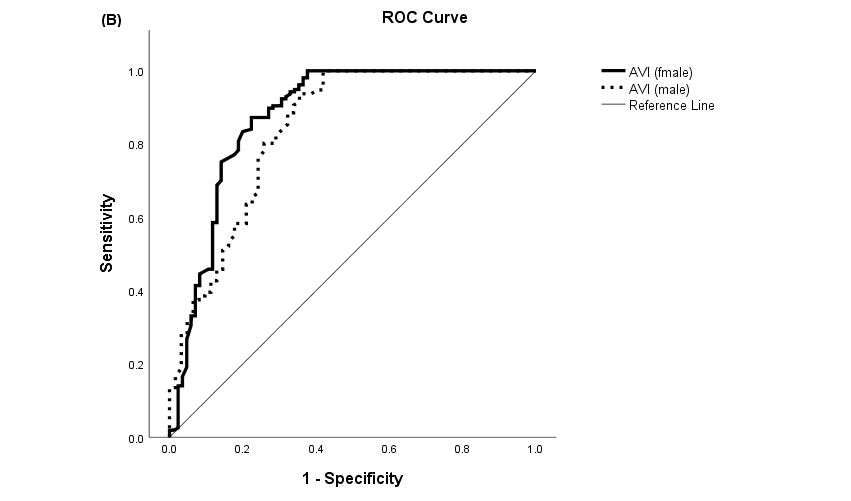


Figure S5: Roc Curve for AVI (A. total with AUC=0.86 (95% CI: 0.82-0.91), B. female with AUC=0.88 (95% CI: 0.82-0.93) and male with AUC=0.84 (95% CI: 0.77-0.91))


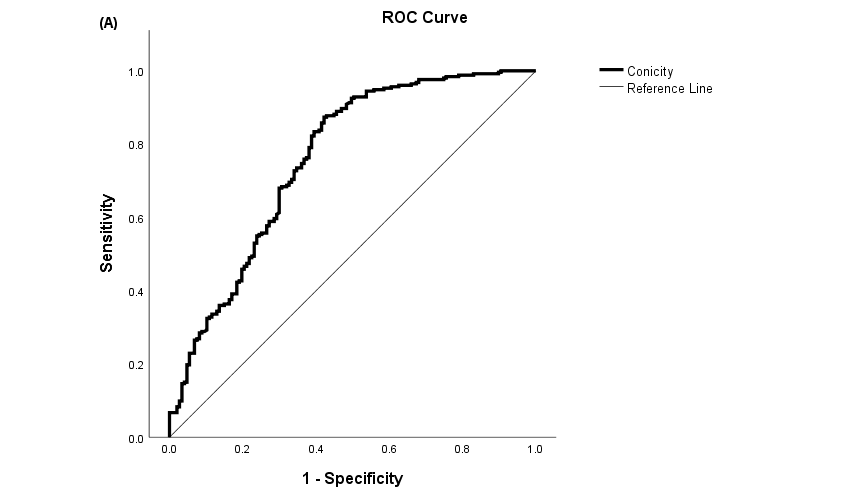


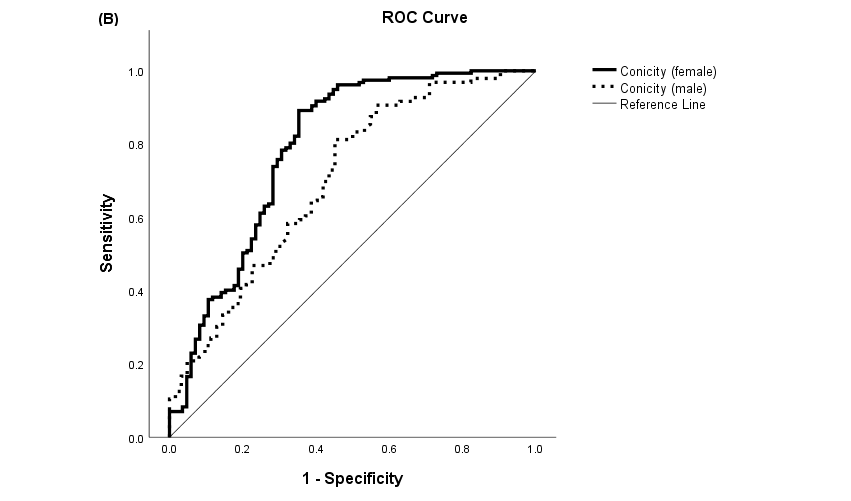


Figure S6: Roc Curve for Conicity (A. total with AUC=0.76 (95% CI: 0.71-0.81), B. female with AUC=0.79 (95% CI: 0.72-0.86) and male with AUC=0.70 (95% CI: 0.62-0.79))


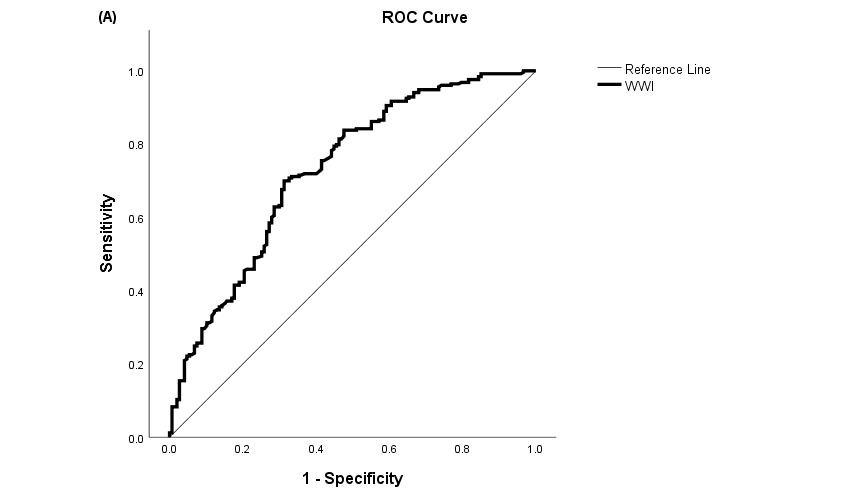


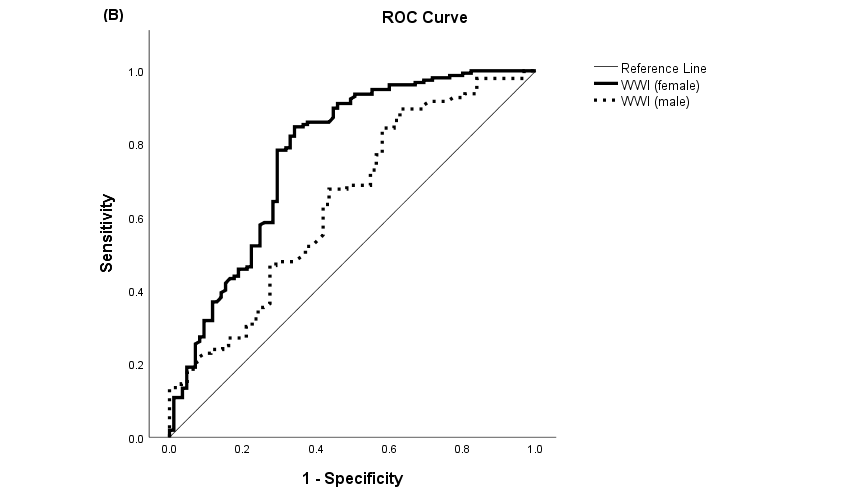


Figure S7: Roc Curve for WWI (A. total with AUC=0.73 (95% CI: 0.68-0.78), B. female with AUC=0.77 (95% CI: 0.71-0.84) and male with AUC=0.64 (95% CI: 0.55-0.73))


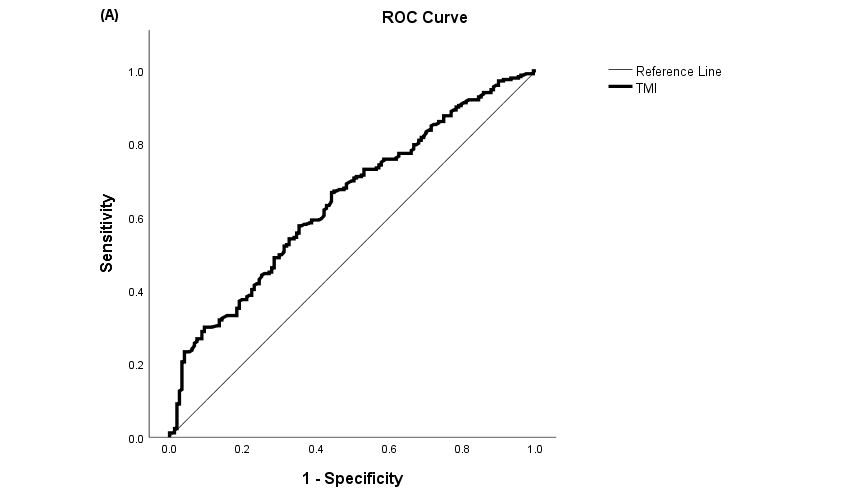


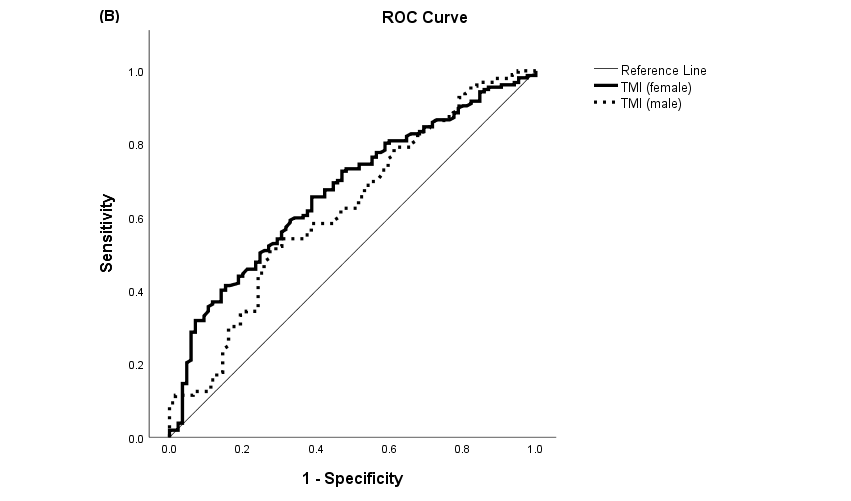


Figure S8: Roc Curve for TMI (A. total with AUC=0.65 (95% CI: 0.59-0.70), B. female with AUC=0.67 (95% CI: 0.60-0.74) and male with AUC=0.62 (95% CI: 0.53-0.71))


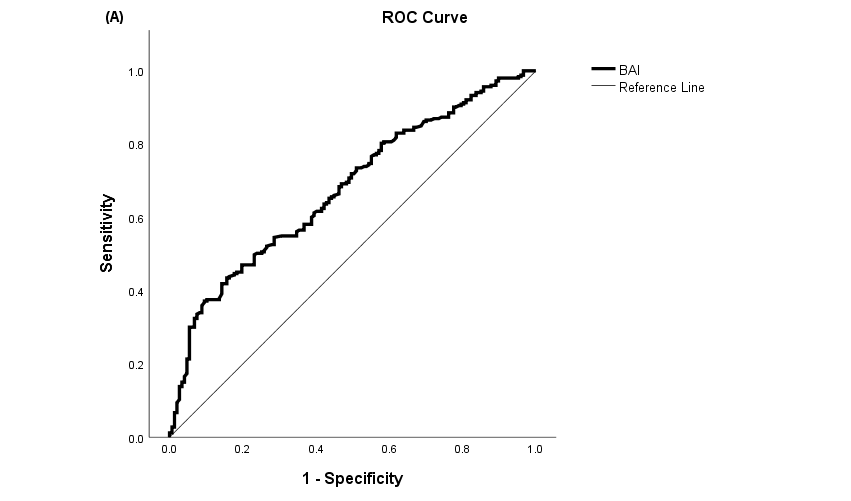


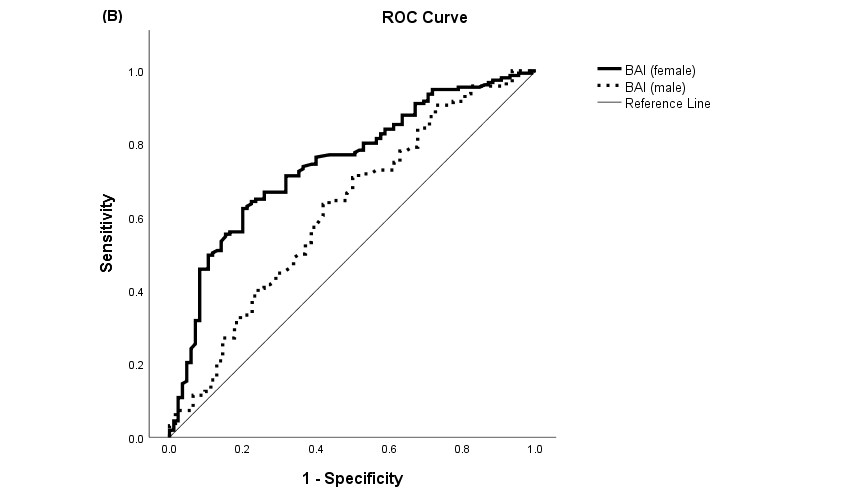


Figure S9: Roc Curve for BAI (A. total with AUC=0.68 (95% CI: 0.62-0.73), B. female with AUC=0.75 (95% CI: 0.68-0.81) and male with AUC=0.62 (95% CI: 0.53-0.71))


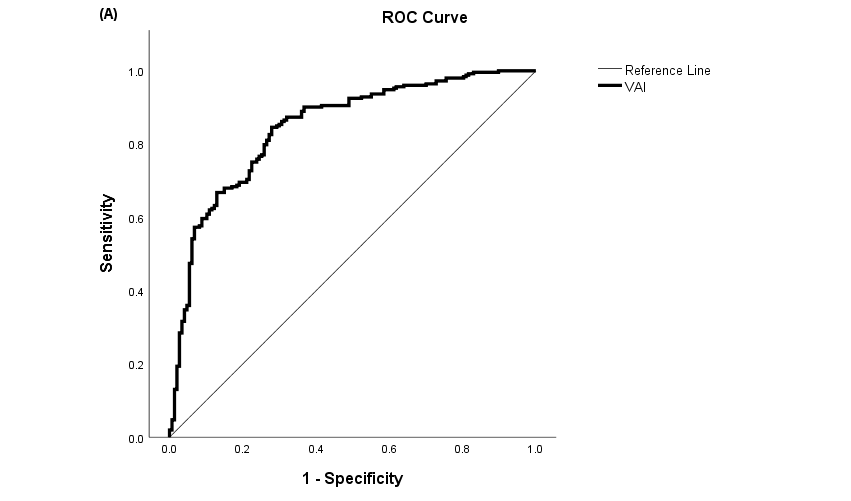


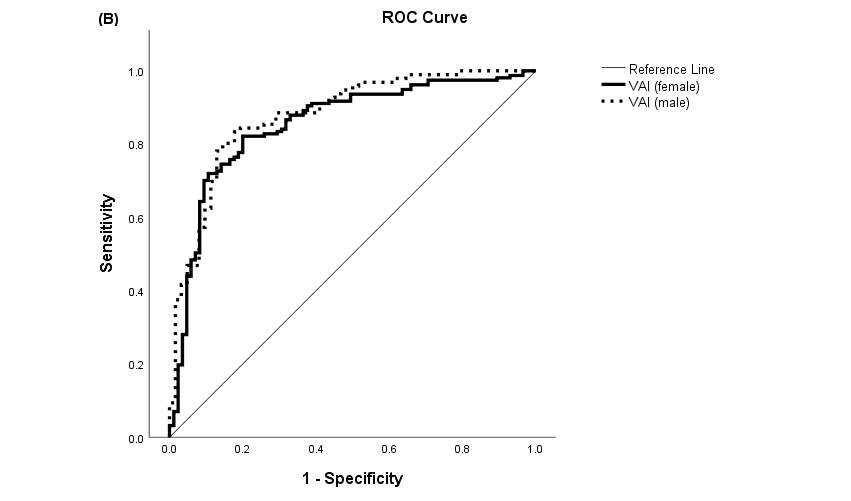


Figure S10: Roc Curve for VAI (A. total with AUC=0.85 (95% CI: 0.81-0.89), B. female with AUC=0.85 (95% CI: 0.80-0.91) and male with AUC=0.88 (95% CI: 0.82-0.93))


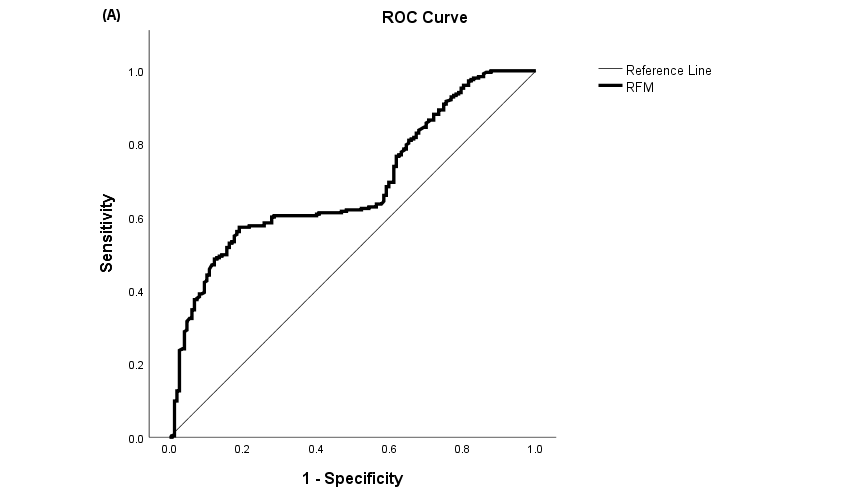


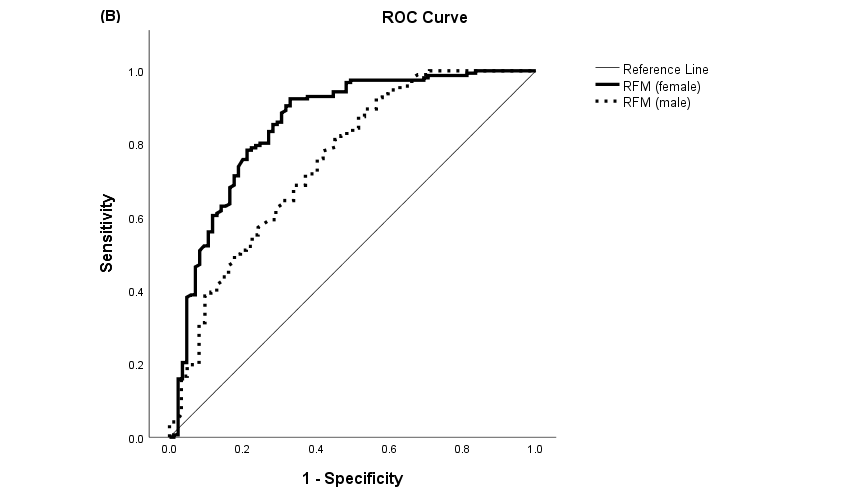


Figure S11: Roc Curve for RFM (A. total with AUC=0.69 (95% CI: 0.64-0.74), B. female with AUC=0.85 (95% CI: 0.80-0.91) and male with AUC=0.75 (95% CI: 0.67-0.83))
